# Climate anxiety impairs sustained attention: objective evidence of a cognitive cost

**DOI:** 10.3389/fpsyg.2025.1624782

**Published:** 2025-10-13

**Authors:** Ekaterina Denkova, Taylor K. Tardibuono, Jason S. Tsukahara, Anthony P. Zanesco, Amishi P. Jha

**Affiliations:** ^1^University of Miami, Coral Gables, FL, United States; ^2^University of Kentucky, Lexington, KY, United States

**Keywords:** eco-anxiety, climate anxiety, cognitive control, cognitive performance, sustained attention

## Abstract

**Introduction:**

As climate anxiety rises globally, it may influence how individuals cognitively engage with the climate crisis. Although cognitive functioning is a key component of climate anxiety, its association with objectively measurable cognitive performance impairment remains unclear. This study examines whether individual differences in climate anxiety correspond with performance on a task measuring sustained attention.

**Methods:**

A total of 182 undergraduate students completed self-report measures of climate anxiety, personal climate change experience, and general anxiety and depression. In addition, they completed the Sustained Attention to Response Task (SART), which measures attentional performance through accuracy and response time variability.

**Results:**

Greater climate anxiety was associated with reduced SART accuracy (*r* = −0.310, *p* < 0.001) and greater response time variability (*r* = 0.188, *p* = 0.024). Consistent with prior research, climate anxiety also correlated with personal experience of climate change and higher levels of anxiety and depression. However, personal experience with climate change as well as anxiety and depression were not significantly related to SART outcomes.

**Discussion:**

Results show that greater climate anxiety is associated with reduced attentional task performance, independent of climate change experience or general psychological distress. Given the central role of attention in decision-making and action-planning, these findings underscore how this cognitive vulnerability may pose a critical barrier to adaptive engagement and effective climate action. The findings also highlight the need for research on approaches to bolster sustained attention as we face growing climate anxiety in a warming world.

## Introduction

Climate change has emerged as an increasingly prominent topic across almost all fields of scientific research over the past decade (Bornmann et al., 2022), largely due to its significant and growing threat to human health (Hayden et al., 2023; Manning and Clayton, 2018; Patz et al., 2014). In addition to its well-established impact on physical health—such as respiratory and cardiovascular conditions—climate change can also have a negative effect on cognitive functioning (Fischer et al., 2024; Chen and Yu, 2024) and psychological health (Clayton, 2020; Clayton and Brown, 2024). One such psychological consequence is the emergence of climate change anxiety, which is characterized by worry and distress in response to the climate crisis (Manning and Clayton, 2018).

The concept of climate anxiety has emerged as a multifaced construct encompassing emotional, cognitive, and behavioral responses to climate change threats (Clayton and Ogunbode, 2023; Chan et al., 2024a; van Valkengoed et al., 2023). While climate anxiety can be seen as a natural reaction to a real and global threat, it can also negatively affect psychological health and interfere with normal daily functioning (Clayton, 2020; Manning and Clayton, 2018). Therefore, as research in this area grows, increasing attention has been directed toward understanding the impact of climate anxiety and delineating its adaptive and maladaptive effects (Brosch, 2021; Clayton, 2020; Taylor, 2020).

Climate anxiety may motivate climate action and pro-environmental behavior (Ballew et al., 2024; Clayton and Parnes, 2025); however, this adaptive effect appears to be influenced by individual differences in emotion regulation, attentional capacities, and personal experiences with climate change (Clayton and Karazsia, 2020; Colombo et al., 2023a, 2023b, 2024; Karl and Stanley, 2024; Heeren et al., 2022; Mathers-Jones and Todd, 2023). Critically, when climate anxiety becomes too elevated, it may impede engagement (Innocenti et al., 2023; Heeren et al., 2022) and negatively affect psychological health (Ogunbode et al., 2022).

Indeed, the maladaptive effects of climate anxiety have been revealed in its association with psychological health issues such as anxiety, depression, and stress (Cosh et al., 2024). The negative link between climate anxiety and well-being appears consistent across diverse populations, with a statistically significant association found in 31 of 32 countries studied (Ogunbode et al., 2022), highlighting the global relevance of this relationship despite national differences in climate anxiety levels (Tam et al., 2023). A meta-analysis of 25 studies further underscores the negative relationship between climate anxiety and psychological health (Gago et al., 2024). While climate anxiety is related to general anxiety and depression, it has been identified as a distinct construct with effects that extend beyond these related conditions (Carlson et al., 2024; Chan et al., 2024a; Schwartz et al., 2023).

Most importantly, longitudinal studies suggest that persistent emotional responses to climate change can, over time, contribute to cognitive-emotional impairments and disruptions in daily functioning (Chan et al., 2024b). In addition, a more recent study has identified the cognitive-emotional component of climate anxiety as a key hub linking climate experiences, worry, pro-environmental behavior, and functional impairments (Heeren et al., 2023). Yet, the evidence for such cognitive-emotional impairments (e.g., difficulty concentrating) relies exclusively on self-report, leaving open the question of whether climate anxiety is associated with *objectively* measurable impairment of cognitive processes.

Among cognitive processes, attention has received growing interest in climate change literature (Carlson et al., 2019), consistent with its well-established role as a core capacity supporting goal-directed behavior (Oberauer, 2024) and complex functions such as emotion regulation, planning, and decision-making (Draheim et al., 2022; Burgoyne and Engle, 2020). However, despite this growing interest, objective investigations of attentional mechanisms remain limited. The few existing studies in this area have predominantly focused on attentional bias, which, in the context of climate change research, refers to the preferential orientation of attention toward climate-relevant stimuli. These studies have examined the association between attentional bias and pro-environmental attitudes and behaviors, but the findings have been inconsistent. While some studies report a positive relationship (Carlson et al., 2019; Meis-Harris et al., 2021), others report a negative association (e.g., Carlson et al., 2020).

Importantly, one study has also considered the role of attentional bias in the link between climate anxiety and pro-environmental behavior. Specifically, the study by Mathers-Jones and Todd (2023) highlighted the importance of variability in attentional bias, rather than bias per se, showing that lower variability (i.e., greater attentional stability) strengthened the association between climate anxiety and pro-environmental behavior. In other words, individuals who were able to maintain more consistent attention to climate-related information were more likely to engage in pro-environmental behavior in response to their anxiety. These findings suggest that the ability to sustain attentional focus over time, beyond initial orienting, may serve as a key mechanism through which anxiety is transformed into constructive action.

Together, this emerging evidence points to the relevance of sustained attention, the ability to maintain focus over time, in understanding adaptive responses to climate anxiety. Yet, no study to date has directly examined the relationship between climate anxiety and objectively measured sustained attention. The present study addresses this gap by investigating whether individual differences in climate anxiety are associated with performance on a sustained attention task while also considering personal experiences with climate change as well as general anxiety and depression, factors that have been previously identified as important and contributing but distinct correlates of climate anxiety (Clayton and Karazsia, 2020; Chan et al., 2024b; Carlson et al., 2024).

## Methods

### Participants

Undergraduate students enrolled in an Introduction to Psychology course at the University of Miami were recruited to participate in this study via the SONA online research participation system during the 2022–2023 academic year. A total of 182 participants (M*_age_* = 19.35, SD*_age_* = 1.46, range = 18–29 years, see Table 1) provided informed consent in accordance with the guidelines of the University of Miami Institutional Review Board (Protocol #20221115). Participants received course credit toward their Introduction to Psychology research participation requirement as compensation.

**Table 1:** Demographics table.

Age	M (SD) = 19.35 (1.46)
Male/Female	
Female	46.70%
Male	52.20%
Other	0.50%
Prefer not to answer	0.50%
Race	
Asian	9.39%
Black or African American	6.63%
Native Hawaiian or Pacific Islander	0.55%
White	71.82%
Other/Not specified	3.87%
Selected more than one category	7.73%
Ethnicity	
Hispanic or Latino	19.20%
Not Hispanic or Latino	67.00%
Other/Not specified	12.10%
Selected more than one category	1.60%
Education	
High school graduate	63.20%
Some college, no degree	31.30%
Occupational, technical, or vocational degree	0.50%
Associates degree	1.10%
Bachelor’s degree (BA, AB, BS, BBA)	3.80%
Activism organization membership	
Yes	3.30%
No	89.60%
N/A	6.00%
Prefer not to answer	1.10%

### Measures

Participants completed a brief battery of online assessments using Inquisit Web (Millisecond Software, LLC), a platform designed for the remote administration of psychological tasks and questionnaires. They were instructed to complete the battery on a desktop or laptop computer, in a quiet, distraction-free environment, and to complete the testing in a single sitting. The testing battery included a series of self-report questionnaires followed by the Sustained Attention to Response Task (SART). The present study focuses specifically on questionnaire measures of climate anxiety, experience of climate change, and symptoms of anxiety and depression described below; additional questionnaires administered are beyond the scope of this report.

*The Climate Change Anxiety Scale* (Clayton and Karazsia, 2020) is a 13-item self-report measure assessing cognitive-emotional distress and functional impairment associated with climate change. Participants rated how often each statement was true for them over the past month (e.g., “Thinking about climate change makes it difficult for me to concentrate”) using a 5-point Likert scale, ranging from 1 (never) to 5 (almost always). Item responses were averaged to create a composite score, with higher scores reflecting greater levels of climate anxiety. The scale measure demonstrated excellent internal consistency (*α* = 0.94) in the current study.

*The Experience of Climate Change* (Clayton and Karazsia, 2020) was assessed via three items (i.e., “I have been directly affected by climate change”; “I know someone who has been directly affected by climate change”; “I have noticed a change in a place that is important to me due to climate change”). Each item was measured using a 5-point Likert scale, ranging from 1 (never) to 5 (almost always). Item responses were averaged, with higher scores reflecting greater experience of climate change. In the current study, the measure demonstrated good internal consistency (α = 0.80).

*The Patient Health Questionnaire* (PHQ4) is a very short 4-item measure used to assess both anxiety and depression in the general population (Kroenke et al., 2009). Participants were asked to rate how often they have been bothered by certain problems (e.g., feeling down) in the past month using a 4-point Likert scale ranging from 0 (not at all) to 3 (nearly every day). All items were summed to obtain a total score, ranging from 0 to 12, with a higher score indicating a higher level of negative psychological health outcomes. In the current study, PHQ4 demonstrated good internal consistency (α = 0.86).

**Figure 1:**
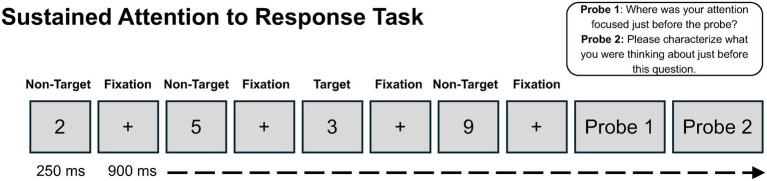
Schematic depiction of the Sustained Attention to Response Task (SART). Digits 0–9 were presented one at a time for 250 ms, followed by a 900 ms fixation. Participants responded to non-target digits and withheld responses to the target digit ‘3’ (5% of trials). Intermittent experience sampling probes assessed mind wandering.

*Sustained Attention to Response Task*. A modified version of the Sustained Attention to Response Task (SART; Robertson et al., 1997) was used to evaluate attentional performance. During SART, single digits (0 through 9) were presented on the screen one at a time for 250 milliseconds, followed by a fixation cross (“+”) for 900 milliseconds (see Figure 1). Participants were instructed to press the spacebar for all digits (non-targets) except the number 3 (target). Responses were recorded during the digit display and the fixation cross. The order of trials was quasi-randomized to ensure that target trials were separated by at least seven non-target trials.

Sets of two consecutive mind-wandering probe questions were interspersed throughout the task. The first probe (Probe 1) asked, “Where was your attention focused just before the probe?” with responses rated on a 5-point scale from 1 (on task) to 5 (off task). The second probe (Probe 2) required participants to classify their thought content into one of seven categories: (1) Focused on the current task, (2) Thoughts about task performance, (3) Distractions from sights/sounds/physical sensations, (4) Negative thoughts unrelated to the task, (5) Positive thoughts unrelated to the task, (6) Neutral thoughts unrelated to the task, and (7) Mind blank. The probes remained on screen until a response was provided.

Participants first completed an 80-trial practice block (with accuracy feedback), which was not included in the analyses. This was followed by two experimental blocks, comprising a total of 592 non-targets, 30 targets, and 30 sets of mind-wandering probe questions. Two measures of objective cognitive performance were obtained from SART. First, accuracy was assessed using A′ (Stanislaw and Todorov, 1999), a sensitivity index that considers both correct hits (correctly withholding responses on target trials) and false alarms (incorrectly withholding responses on non-target trials). Higher A′ scores indicate better discrimination between target and non-target stimuli and thus greater sustained attention. Second, response time (RT) variability was indexed by the intra-individual coefficient of variation (ICV), calculated as the standard deviation of RTs for correct non-target trials divided by the mean RT for those trials, providing a measure of attentional stability. The higher the ICV, the greater the response time variability and the more frequent the lapses in sustained attention; lower ICV indicates more stable attention (Bastian and Sackur, 2013; Zanesco et al., 2024).

### Analyses

For self-report questionnaires, participants with incomplete responses on one or more items were excluded from analyses involving that specific measure. SART data were excluded based on the following criteria: (i) missing or incomplete task data, such as task failures or early termination (*n* = 12); (ii) insufficient task engagement, operationalized as failure to respond on more than two-thirds of trials (*n* = 14); and (iii) performance at or below chance level, indicated by an A′ score < 0.50 (*n* = 12). No additional exclusions were applied to the SART dataset.

To examine relationships between variables of interest, a series of bivariate Pearson correlations were conducted using IBM SPSS Statistics (Version 29).

## Results

Descriptive statistics and correlation results are reported in Table 2. Additional analyses, including results for climate anxiety subscales as well as anxiety and depression PHQ4 subscales are provided in the Supplementary material.

**Table 2:** Descriptive and correlation statistics.

Variables	Descriptives							Correlations				
	*N*	Mean	SD	Range		Skew	Kurt	1	2	3	4	5
1. Climate anxiety	178	1.338	0.522	1.000	3.154	1.808	2.532	–	0.399**	0.208**	−0.310**	0.188*
2. Experience of climate change	180	1.811	0.922	1.000	5.000	1.259	1.124	178	–	0.177*	0.010	−0.066
3. Anxiety and depression (PHQ4)	179	4.160	3.044	0.000	12.000	0.631	−0.331	177	179	–	0.090	−0.099
4. SART A’	144	0.785	0.114	0.500	0.983	−0.514	−0.399	143	144	144	–	−0.822**
5. SART ICV	144	0.513	0.230	0.148	1.173	0.614	−0.380	143	144	144	144	–

For the correlation section of the table, the lower triangle is the count, and the upper triangle is the correlation coefficient. *p < 0.05, **p < 0.01.

**Figure 2:**
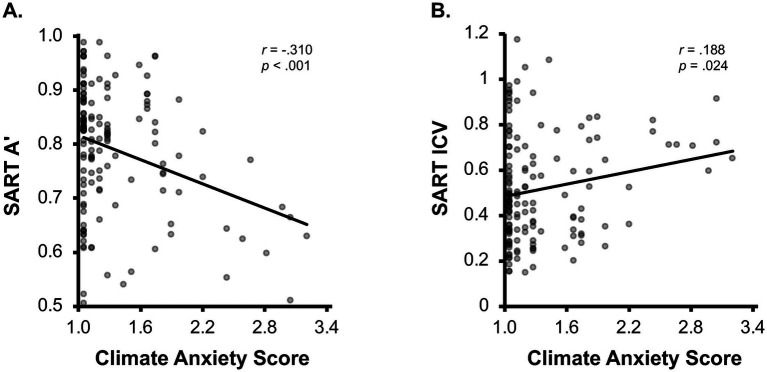
Scatterplots depicting the relationship between climate change anxiety scores. **(A)** SART A’ and **(B)** SART ICV.

Correlation analyses revealed that climate anxiety scores were significantly negatively correlated with SART A′, *r* = −0.310, *p* < 0.001 (Figure 2A), and significantly positively correlated with SART ICV, *r* = 0.188, *p* = 0.024 (Figure 2B). Experience of climate change and PHQ scores were not significantly correlated with SART outcomes (*p*-values > 0.2). Consistent with the existing literature, climate anxiety scores were positively correlated with both experience of climate change, *r* = 0.399, *p* < 0.001, and PHQ scores, *r* = 0.208, *p* = 0.005.

The present results indicate that individuals with higher levels of climate anxiety not only report greater symptoms of general anxiety and depression, but also exhibit reduced objective attentional performance, as evidenced by lower accuracy and increased response time variability, which were not significantly related to the experience of climate change and overall psychological distress.

## Discussion

The present study investigated the impact of climate anxiety on sustained attention, assessed via the Sustained Attention to Response Task (SART), utilizing an individual differences approach. Correlational analyses indicated that elevated climate anxiety was significantly associated with not only increased anxiety and depression symptoms but also poorer attentional performance, as evidenced by reduced SART A′ scores and greater response time variability (SART ICV). Notably, neither experience with climate change nor general anxiety and depression were significantly related to SART outcomes.

These findings extend prior research by demonstrating that heightened climate anxiety is associated not only with increased anxiety and depression levels but, critically, with objective impairments in cognitive functioning. Although few studies have utilized cognitive tasks in the climate change research (e.g., Carlson et al., 2019, 2020, 2022; Meis-Harris et al., 2021), these investigations predominantly focused on attentional bias toward climate-related pictures and their impact on climate action. In contrast, the present study directly assessed the relationship between climate anxiety and objectively measured cognitive functioning. This study thus advances the climate anxiety literature by demonstrating that higher levels of climate anxiety are significantly associated with diminished objective attentional performance.

Importantly, stability of attention, examined in the context of attentional bias research (Mathers-Jones and Todd, 2023) has been identified as a key factor in transforming climate anxiety into climate action. Yet the present findings suggest that higher climate anxiety is associated with reduced sustained attention. This raises a concerning paradox: the very cognitive process proposed to facilitate adaptive engagement with the climate crisis (i.e., sustained attention) may be weakened or less effective in individuals experiencing climate anxiety.

One potential avenue for addressing this paradox involves interventions aimed at enhancing sustained attention. Among such interventions, mindfulness training has shown particular promise. A growing body of evidence supports the efficacy of mindfulness training in enhancing cognitive functioning, even in healthy, non-clinical populations. Meta-analyses have consistently shown that mindfulness training improves cognitive performance, particularly on tasks involving sustained attention (Yakobi et al., 2021; Verhaeghen, 2021; Zainal and Newman, 2024). Mindfulness training has also been shown to enhance psychological well-being (Galante et al., 2023), with some emerging studies suggesting that these benefits may, in part, stem from improvements in cognitive functioning (Jha et al., 2019; Roca et al., 2023). Taken together, this evidence suggests that mindfulness training may offer a promising approach for supporting cognitive resilience in individuals experiencing elevated levels of climate anxiety (Ikiz and Carlson, 2025).

While the present study offers valuable insights into the relationship between climate anxiety and cognitive performance, there are some limitations. First, the relatively small sample size (*N* = 182), composed primarily of undergraduate psychology students, limits the generalizability of the findings despite statistically significant results. Replicating findings in a larger and more diverse sample in future studies would offer stronger support and enhance the applicability of the current findings. Second, the average climate anxiety scores (*M* = 1.34) were relatively lower than some prior findings (e.g., *M* = 2.04; Maduneme, 2024) but aligned with others (*M* = 1.41–1.67; Schwartz et al., 2023). Nonetheless, climate anxiety was significantly linked to poorer attentional performance and higher anxiety and depression, consistent with past research (Cosh et al., 2024). A third potential limitation of the present study is the absence of clinical screening for factors known to influence cognitive performance, such as traumatic brain injury, neurodevelopmental or psychiatric conditions (e.g., ADHD), and psychotropic medication use. While SART exclusion criteria were applied to help ensure task engagement and to minimize the inclusion of participants with extremely low performance, future studies should consider additional clinical screening. Additionally, the study used a single cognitive task, the SART, to measure cognitive performance. While this task sheds light on sustained attention, future research would benefit from including a wider variety of cognitive measures to capture attentional control and better understand the relationship between climate anxiety and cognition. Finally, the study did not account for pro-environmental behavior, which could offer a more complete picture of how climate anxiety, cognitive performance, and active engagement in climate action are interrelated.

## Conclusions and future direction

The present study found that higher levels of climate anxiety are associated with poorer cognitive functioning in a sample of young adults. These findings contribute to the growing literature on climate anxiety by helping to delineate its adaptive and maladaptive effects. By objectively measuring cognitive performance, the study suggests that reduced sustained attention is associated with elevated climate anxiety, but not personal experience with climate change or levels of general anxiety and depression.

Future research should aim to clarify the direction and nature of the relationship between sustained attention and climate anxiety, as well as how this relationship influences behavior. Given that attention is a core cognitive capacity that supports both emotion regulation and goal-directed behavior (Burgoyne and Engle, 2020; Oberauer, 2024), identifying how sustained attention and climate anxiety interact is critical for understanding their impact on daily functioning. To examine potential causal pathways, experimental studies could manipulate climate-related anxiety, for example by exposing participants to climate change-related stimuli and assess immediate effects on sustained attention performance. Alternatively, studies could deplete attentional resources using cognitive load tasks and examine whether individuals exhibit increased climate anxiety in response to climate-related cues. Such studies would help determine whether the relationship between sustained attention and climate anxiety is bidirectional, unidirectional, or context-dependent, offering a stronger foundation for designing targeted solutions.

## Data Availability

The original contributions presented in the study are included in the article/Supplementary material, further inquiries can be directed to the corresponding author.

## References

[ref1] Ballew M. T. Uppalapati S. S. Myers T. Carman J. Campbell E. Rosenthal S. A.. (2024). Climate change psychological distress is associated with increased collective climate action in the U.S. npj Climate Action 3:8. doi: 10.1038/s44168-024-00172-8, PMID: 40610540

[ref2] Bastian M. Sackur J. (2013). Mind wandering at the fingertips: automatic parsing of subjective states based on response time variability. Front. Psychol. 4:573. doi: 10.3389/fpsyg.2013.00573, PMID: 24046753 PMC3763218

[ref3] Bornmann L. Haunschild R. Boyack K. Marx W. Minx J. C. (2022). How relevant is climate change research for climate change policy? An empirical analysis based on Overton data. PLoS One 17:e0274693. doi: 10.1371/journal.pone.0274693, PMID: 36137101 PMC9499296

[ref4] Brosch T. (2021). Affect and emotions as drivers of climate change perception and action: a review. Curr. Opin. Behav. Sci. 42, 15–21. doi: 10.1016/j.cobeha.2021.02.001

[ref5] Burgoyne A. P. Engle R. W. (2020). Attention control: a cornerstone of higher-order cognition. Curr. Dir. Psychol. Sci. 29, 624–630. doi: 10.1177/0963721420969371

[ref6] Carlson J. M. Fang L. Coughtry-Carpenter C. Foley J. (2022). Reliability of attention bias and attention bias variability to climate change images in the dot-probe task. Front. Psychol. 13:1021858. doi: 10.3389/fpsyg.2022.1021858, PMID: 36710831 PMC9878553

[ref7] Carlson J. M. Foley J. Fang L. (2024). Climate change on the brain: neural correlates of climate anxiety. J. Anxiety Disord. 103:102848. doi: 10.1016/j.janxdis.2024.102848, PMID: 38431988

[ref8] Carlson J. M. Kaull H. Steinhauer M. Zigarac A. Cammarata J. (2020). Paying attention to climate change: positive images of climate change solutions capture attention. J. Environ. Psychol. 71:101477. doi: 10.1016/j.jenvp.2020.101477

[ref9] Carlson J. M. Lehman B. R. Thompson J. L. (2019). Climate change images produce an attentional bias associated with pro-environmental disposition. Cogn. Process. 20, 385–390. doi: 10.1007/s10339-019-00902-5, PMID: 30671678

[ref10] Chan H. W. Lin L. Tam K. P. Hong Y. Y. (2024b). From negative feelings to impairments: a longitudinal study on the development of climate change anxiety. J. Anxiety Disord. 107:102917. doi: 10.1016/j.janxdis.2024.102917, PMID: 39217778

[ref11] Chan H.-W. Tam K.-P. Clayton S. (2024a). Testing an integrated model of climate change anxiety. J. Environ. Psychol. 97:102368. doi: 10.1016/j.jenvp.2024.102368

[ref12] Chen H. Yu Y. (2024). Does climate change exacerbate gender inequality in cognitive performance? Glob. Environ. Chang. 89:102941. doi: 10.1016/j.gloenvcha.2024.102941

[ref13] Clayton S. (2020). Climate anxiety: psychological responses to climate change. J. Anxiety Disord. 74:102263. doi: 10.1016/j.janxdis.2020.102263, PMID: 32623280

[ref14] Clayton S. Brown L. A. (2024). Climate change and mental health. JAMA 331, 1761–1762. doi: 10.1001/jama.2024.1839, PMID: 38691377 PMC11343074

[ref15] Clayton S. Karazsia B. T. (2020). Development and validation of a measure of climate change anxiety. J. Environ. Psychol. 69:101434. doi: 10.1016/j.jenvp.2020.101434

[ref16] Clayton S. Ogunbode C. (2023). Looking at emotions to understand responses to environmental challenges. Emot. Rev. 15, 275–278. doi: 10.1177/17540739231193757

[ref17] Clayton S. Parnes M. F. (2025). Anxiety and activism in response to climate change. Curr. Opin. Psychol. 62:101996. doi: 10.1016/j.copsyc.2025.101996, PMID: 39889454

[ref18] Colombo S. L. Chiarella S. G. Lefrançois C. Fradin J. Simione L. Raffone A. (2023a). Probing pro-environmental behaviour: a systematic review on its relationship with executive functions and self-regulation processes. J. Environ. Psychol. 92:102153. doi: 10.1016/j.jenvp.2023.102153

[ref19] Colombo S. L. Chiarella S. G. Raffone A. Simione L. (2023b). Understanding the environmental attitude-behaviour gap: the moderating role of dispositional mindfulness. Sustain. For. 15:7285. doi: 10.3390/su15097285

[ref20] Colombo S. L. Raffone A. Simione L. (2024). On the relationship between climate change anxiety and pro-environmental behaviour: dispositional mindfulness as a double-edged sword. Mindfulness 16, 366–380. doi: 10.1007/s12671-024-02483-7

[ref21] Cosh S. M. Ryan R. Fallander K. Robinson K. Tognela J. Tully P. J.. (2024). The relationship between climate change and mental health: a systematic review of the association between eco-anxiety, psychological distress, and symptoms of major affective disorders. BMC Psychiatry 24:833. doi: 10.1186/s12888-024-06274-1, PMID: 39567913 PMC11577747

[ref22] Draheim C. Pak R. Draheim A. A. Engle R. W. (2022). The role of attention control in complex real-world tasks. Psychon. Bull. Rev. 29, 1143–1197. doi: 10.3758/s13423-021-02052-2, PMID: 35167106 PMC8853083

[ref23] Fischer S. Naegeli K. Cardone D. Filippini C. Merla A. Hanusch K. U.. (2024). Emerging effects of temperature on human cognition, affect, and behaviour. Biol. Psychol. 189:108791. doi: 10.1016/j.biopsycho.2024.108791, PMID: 38599369

[ref24] Gago T. Sargisson R. J. Milfont T. L. (2024). A meta-analysis on the relationship between climate anxiety and wellbeing. J. Environ. Psychol. 94:102230. doi: 10.1016/j.jenvp.2024.102230

[ref25] Galante J. Friedrich C. Collaboration of Mindfulness, T Dalgleish T. Jones P. B. White I. R.. (2023). Individual participant data systematic review and meta-analysis of randomised controlled trials assessing adult mindfulness-based programmes for mental health promotion in non-clinical settings. Nat. Ment. Health 1, 462–476. doi: 10.1038/s44220-023-00081-5, PMID: 37867573 PMC7615230

[ref26] Hayden M. H. Schramm P. J. Beard C. B. Bell J. E. Bernstein A. S. Bieniek-Tobasco A.. (2023). “Human health” in Fifth National Climate Assessment. eds. Crimmins A. R. Avery C. W. Easterling D. R. Kunkel K. E. Stewart B. C. Maycock T. K. (Washington, DC: U.S. Global Change Research Program).

[ref27] Heeren A. Mouguiama-Daouda C. Contreras A. (2022). On climate anxiety and the threat it may pose to daily life functioning and adaptation: a study among European and African French-speaking participants. Clim. Chang. 173:15. doi: 10.1007/s10584-022-03402-2, PMID: 35912274 PMC9326410

[ref28] Heeren A. Mouguiama-Daouda C. McNally R. J. (2023). A network approach to climate change anxiety and its key related features. J. Anxiety Disord. 93:102625. doi: 10.1016/j.janxdis.2022.102625, PMID: 36030121

[ref30] Ikiz B. Carlson J. M. (2025). Neural pathways to resilience: leveraging neuroscience to understand and mitigate eco-anxiety. Ann. N. Y. Acad. Sci. 1547, 18–23. doi: 10.1111/nyas.15347, PMID: 40214627

[ref31] Innocenti M. Santarelli G. Lombardi G. S. Ciabini L. Zjalic D. Di Russo M.. (2023). How can climate change anxiety induce both pro-environmental Behaviours and eco-paralysis? The mediating role of general self-efficacy. Int. J. Environ. Res. Public Health 20:3085. doi: 10.3390/ijerph20043085, PMID: 36833780 PMC9960236

[ref9001] Jha A. P. Denkova E. Zanesco A. P. Witkin J. E. Rooks J. Rogers S. L. (2019). Does mindfulness training help working memory ‘work’ better?. Curr Opin Psychol, 28, 273–278. doi: 10.1016/j.copsyc.2019.02.012 30999122

[ref32] Karl J. A. Stanley S. K. (2024). Is mindfulness a double-edged sword? Associations with climate anxiety and pro-environmental behavior. Mindfulness 15, 2207–2217. doi: 10.1007/s12671-024-02427-1

[ref33] Kroenke K. Spitzer R. L. Williams J. B. Lowe B. (2009). An ultra-brief screening scale for anxiety and depression: the PHQ-4. Psychosomatics 50, 613–621. doi: 10.1176/appi.psy.50.6.613, PMID: 19996233

[ref34] Maduneme E. (2024). Some slice of climate anxiety… Is good: a cross-sectional survey exploring the relationship between college students media exposure and perceptions about climate change. J. Health Commun. 29, 45–56. doi: 10.1080/10810730.2024.2354370, PMID: 38775847

[ref35] Manning C. Clayton S. (2018). “Threats to mental health and wellbeing associated with climate change” in Psychology and climate change, 217–244. doi: 10.1016/B978-0-12-813130-5.00009-6

[ref36] Mathers-Jones J. Todd J. (2023). Ecological anxiety and pro-environmental behaviour: the role of attention. J. Anxiety Disord. 98:102745. doi: 10.1016/j.janxdis.2023.102745, PMID: 37480627

[ref37] Meis-Harris J. Eyssel F. Kashima Y. (2021). Are you paying attention? How pro-environmental tendencies relate to attentional processes. J. Environ. Psychol. 74:101591. doi: 10.1016/j.jenvp.2021.101591

[ref38] Oberauer K. (2024). The meaning of attention control. Psychol. Rev. 131, 1509–1526. doi: 10.1037/rev0000514, PMID: 39418416

[ref39] Ogunbode C. A. Doran R. Hanss D. Ojala M. Salmela-Aro K. van den Broek K. L.. (2022). Climate anxiety, wellbeing and pro-environmental action: correlates of negative emotional responses to climate change in 32 countries. J. Environ. Psychol. 84:101887. doi: 10.1016/j.jenvp.2022.101887

[ref40] Patz J. A. Frumkin H. Holloway T. Vimont D. J. Haines A. (2014). Climate change: challenges and opportunities for global health. JAMA 312, 1565–1580. doi: 10.1001/jama.2014.13186, PMID: 25244362 PMC6108836

[ref41] Robertson I. H. Manly T. Andrade J. Baddeley B. T. Yiend J. (1997). 'Oops!': performance correlates of everyday attentional failures in traumatic brain injured and normal subjects. Neuropsychologia 35, 747–758. doi: 10.1016/S0028-3932(97)00015-8, PMID: 9204482

[ref9002] Roca P. Vazquez C. Diez G. McNally R. J. (2023). How do mindfulness and compassion programs improve mental health and well-being? The role of attentional processing of emotional information. J Behav Ther Exp Psychiatry 81, 101895–758. doi: 10.1016/j.jbtep.2023.101895, PMID: 37515955

[ref42] Schwartz S. E. O. Benoit L. Clayton S. Parnes M. F. Swenson L. Lowe S. R. (2023). Climate change anxiety and mental health: environmental activism as buffer. Curr. Psychol. 42, 16708–16721. doi: 10.1007/s12144-022-02735-6, PMID: 35250241 PMC8883014

[ref43] Stanislaw H. Todorov N. (1999). Calculation of signal detection theory measures. Behav. Res. Methods Instrum. Comput. 31, 137–149. doi: 10.3758/Bf03207704 10495845

[ref44] Tam K.-P. Chan H.-W. Clayton S. (2023). Climate change anxiety in China, India, Japan, and the United States. J. Environ. Psychol. 87:101991. doi: 10.1016/j.jenvp.2023.101991

[ref45] Taylor S. (2020). Anxiety disorders, climate change, and the challenges ahead: introduction to the special issue. J. Anxiety Disord. 76:102313. doi: 10.1016/j.janxdis.2020.102313, PMID: 32992267 PMC7507977

[ref46] van Valkengoed A. M. Steg L. de Jonge P. (2023). Climate anxiety: a research agenda inspired by emotion research. Emot. Rev. 15, 258–262. doi: 10.1177/17540739231193752

[ref47] Verhaeghen P. (2021). Mindfulness as attention training: meta-analyses on the links between attention performance and mindfulness interventions, long-term meditation practice, and trait mindfulness. Mindfulness 12, 564–581. doi: 10.1007/s12671-020-01532-1

[ref49] Yakobi O. Smilek D. Danckert J. (2021). The effects of mindfulness meditation on attention, executive control and working memory in healthy adults: a meta-analysis of randomized controlled trials. Cogn. Ther. Res. 45, 543–560. doi: 10.1007/s10608-020-10177-2

[ref50] Zainal N. H. Newman M. G. (2024). Mindfulness enhances cognitive functioning: a meta-analysis of 111 randomized controlled trials. Health Psychol. Rev. 18, 369–395. doi: 10.1080/17437199.2023.2248222, PMID: 37578065 PMC10902202

[ref51] Zanesco A. P. Denkova E. Barry J. Jha A. P. (2024). Mind wandering is associated with worsening attentional vigilance. J. Exp. Psychol. Hum. Percept. Perform. 50, 1049–1066. doi: 10.1037/xhp0001233, PMID: 39172363

