# Supplementary material for: Climate anxiety impairs sustained attention: objective evidence of a cognitive cost
Source: Front Psychol. 2025 Oct 13;16:1624782. doi: 10.3389/fpsyg.2025.1624782 (PMC12557577; doi:10.3389/fpsyg.2025.1624782)
Supplement: Supplementary file 2 [file Table_1.PDF]

Supplemental Materials

Sub-scales description

*Cognitive-Emotional Impairment (*Clayton & Karazsia, 2020) is an 8-item self-report subscale of the Climate Change Anxiety Scale that assess cognitive-emotional distress associated with climate change. Participants rated how often each statement was true for them over the past month (e.g., "Thinking about climate change makes it difficult for me to sleep") using a 5-point Likert scale, ranging from 1 (never) to 5 (almost always). Item responses were averaged to create a composite score, with higher scores reflecting greater levels of climate-related cognitive-emotional impairment. In the present study, the measure demonstrated good internal consistency ($\alpha = .91$).

Functional Impairment (Clayton & Karazsia, 2020) is a 5-item self-report subscale of the Climate Change Anxiety Scale that assesses functional impairment associated with climate change. Participants rated how often each statement was true for them over the past month (e.g., "My concerns about climate change undermine my ability to work to my potential") using a 5-point Likert scale, ranging from 1 (never) to 5 (almost always). Item responses were averaged to create a composite score, with higher scores reflecting greater levels of climate-related functional impairment. Additionally, the measure demonstrated good internal consistency, ($\alpha = .85$).

Anxiety (PHQ4) is a very short 2-item subscale of the PHQ4 used to assess anxiety in the general population (Kroenke et al., 2009). Participants were asked to rate how often they have been bothered by certain problems (e.g., feeling on edge) in the past month using a 4-point Likert scale ranging from 0 (not at all) to 3 (nearly every day). All items were summed to obtain a total score, ranging from 0 to 6, with a higher score indicating a higher level of anxiety. Additionally, in the present study, the measure demonstrated good internal consistency ($\alpha = .85$).

Depression (PHQ4) is a very short 2-item subscale of the PHQ4 used to assess depression in the general population (Kroenke et al., 2009). Participants were asked to rate how often they have been bothered by certain problems (e.g., feeling hopeless) in the past month using a 4-point Likert scale ranging from 0 (not at all) to 3 (nearly every day). All items were summed to obtain a total score, ranging from 0 to 6, with a higher score indicating a higher level of depression. Additionally, in the present study, the measure demonstrated good internal consistency (α = .81).

Results

Supplemental Table. Descriptive and Correlation Statistics with sub-scales

Variables	Descriptives							Correlations						
	N	Mean	SD	Range		Skew	Kurt	1	2	3	4	5	6	7
1. CA: Cognitive Imapirement	178	1.350	0.556	1.000	3.250	1.737	2.024	-	.798**	.384**	.182*	.179*	-.297**	.185*
2. CA: Functional Impairement	179	1.317	0.537	1.000	3.600	2.011	3.629	178	-	.373**	.165*	.190*	-.288**	.165*
3. Experience of Climate Change	180	1.811	0.922	1.000	5.000	1.259	1.124	178	179	-	.166*	.153*	.010	-.066
4. Anxiety	180	2.490	1.795	0.000	6.000	0.536	-0.644	178	179	180	-	.635**	.059	-.081
5. Depression	179	1.660	1.565	0.000	6.000	0.847	0.171	177	178	179	179	-	.104	-.096
6. SART A'	144	0.785	0.114	0.500	0.983	-0.514	-0.399	143	144	144	144	144	-	-.822**
7. SART ICV	144	0.513	0.230	0.148	1.173	0.614	-0.380	143	144	144	144	144	144	-

Note: For the correlation section of the table, the lower triangle is the count, and the upper triangle is the correlation coeffieicent. * $p < .05$, ** $p < .01$, *** $p < .001$
